# Induction of Antioxidant Protein HO-1 Through Nrf2-ARE Signaling Due to Pteryxin in *Peucedanum Japonicum* Thunb in RAW264.7 Macrophage Cells

**DOI:** 10.3390/antiox8120621

**Published:** 2019-12-05

**Authors:** Junsei Taira, Takayuki Ogi

**Affiliations:** 1 Department Bioresources Engendering, Okinawa College, National Institute of Technology, Okinawa 905-2192, Japan; 2 Department of Environment and Natural Resources, Okinawa Industrial Technology Center, Okinawa 904-2234, Japan; ogitkyuk@pref.okinawa.lg.jp

**Keywords:** coumarin, pteryxin, HO-1, Nrf2, oxidative stress, *Peucedanum japonicum* Thunb, RAW264.7 cells

## Abstract

This study focused on exploring the nuclear factor-erythroid-2-related factor (Nrf2) active compound to avoid oxidative stress related to various diseases, such as obesity and diabetes mellitus. The activity of the Nrf2-ARE (antioxidant response element) signaling was evaluated by a reporter assay involving over five hundred various edible medicinal herbs, and the highest Nrf2 activity was found in the ethanol extract of *Peucedanum japonicum* leaves. The active compound in the extract was isolated by high performance liquid chromatography (HPLC), and the chemical structure was identical to pteryxin based on ^1^H, ^13^C-NMR spectra and liquid chromatography/time-of-fright mass spectrometer (LC/TOF/MS). From the pteryxin, the transcription factor Nrf2 was accumulated in the nucleus and resulted in the expression of the antioxidant protein, heme oxygenase-1 (HO-1). In addition, the Nrf2 activity involving HO-1 expression due to coumarin derivatives was evaluated together with pteryxin. This suggested that the electrophilicity, due to the α,β-carbonyl and/or substituted acyl groups in the molecule, modulates the cysteine residue in Keap1 via the Michel reaction, at which point the Nrf2 is dissociated from the Keap1. These results suggest that pteryxin will be a useful agent for developing functional foods.

## 1. Introduction

Some *Peucedanum* species belonging to the *Apiaceae* family contain therapeutic properties and are used in traditional medicine against various conditions, including sore throats, coughs, colds, and headaches [1]. A species of *Peucedanum japonicum* Thunb has been used as a folk medicine in Japan, Taiwan, and China, and the antioxidant and antityrosinase active compounds were found in the leaf extract of the *Peucedanum* species [2,3]. Recent studies have demonstrated that the ethanol (EtOH) extract of *P. japonicum* has an anti-obesity effect and that it contains coumarin-related compounds that the affect diabetes and obesity, both of which are bioaccessible to the systemic tissues [4,5,6,7,8,9].

Oxidative stress, with the excess production of reactive oxygen species (ROS), is related to an increased risk of developing several diseases, including obesity and diabetes mellitus. The ROS and reactive nitrogen species (RNS), due to the oxidative stress in the cells, induce antioxidant enzymes such as SOD, glutathione peroxidase, and thioredoxin (Trx) as the first line of defense. The Nrf2 (Nuclear factor-erythroid-2-related factor)-ARE (antioxidant response element) signaling responds to the cell damage with the excess production of ROS and RNS or electrophiles. The Nrf2 dissociates from the Kelch-like ECH-associated protein 1 (Keap1) by electrophiles and the oxidative stress, which regulates the expression of the ARE region containing phase II detoxifying/antioxidant enzymes, such as glutathione *S*-transferase, NAD(P)H quinone oxidoreductase-1, Trx, and heme oxygenase-1 (HO-1) [10]. The Nrf2 plays a significant role in the regulation of adipocyte differentiation, obesity, and insulin resistance [11]. Certain dietary chemopreventive agents target the Keap1 by oxidizing or chemically modifying its specific cysteine thiols, which can induce ARE-mediated genes expression [12,13]. In our previous studies, it was demonstrated that marine natural products modulate the HO-1 protein expression through Nrf2 activation in both normal cells and cancer cells [14,15].

This study assessed the Nrf2 activity in various edible medicinal plants, and the highest Nrf2 activity was found in the EtOH extract of *P. japonicum* leaves. In addition, this study shows that pteryxin was the active compound in the extract, which was enhanced by the HO-1 protein expression through the Nrf2-ARE signaling.

## 2. Materials and Methods

### 2.1. Materials

Coumarin and 3,4-dihydrocoumarin were purchased from the FUJI Firm Wako Pure Chemical Corporation (Osaka, Japan) and pyranocoumarin was obtained from Sigma-Aldrich Co. LLC (St. Louis, USA). The products of the antibodies, such as anti-Nrf2 (Santa Cruz Biotechnology, Inc., TX, USA), anti-HO-1 (StressMarq Biosciences, Inc., Victoria, Canada), and anti-β-actin (FUJI Firm Wako Pure Chemical Corporation) were used for detecting the protein expressions. The cytotoxicity was determined using 3-(4,5-dimethyl-2-thiazlyl)-2,5-diphenyltetrazolium bromide (MTT, FUJI Firm Wako Pure Chemical Corporation).

### 2.2. Isolation of Pteryxin

Pteryxin was isolated from the dried-leaf powder of *P. japonicum*. The dried-leaf powder of *P. japonicum* (20 g) was extracted by 50% EtOH (210 mL) using a Dionex ASE 350 accelerated solvent extractor (Thermo Fisher Scientific, Inc., Waltham, MA, USA). The extract was loaded on a Diaion HP20 column (100 × 20 mm I.D., Mitsubishi Rayon Aqua Solutions Co. Ltd., Tokyo, Japan), then the sample was sequentially eluted, each with 100 mL of 50% and absolute EtOH. The EtOH fraction was evaporated in vacuo, and the residue (325 mg) was separated by centrifugal partition chromatography (Easy-PREPccc, 318 mL of coil column, Kutuwa Sangyo, Hiroshima, Japan) in the two-phase solvent system of n-hexane/chloroform/70% methanol (9:1:10 in *v/v/v*). Its lower layer (mobile phase) was separated at 3.0 mL/min and 1110 rpm. The collected fractions (31.8 mg) were purified on a reversed-phase chromatographic column (XBridge C18 column, 150 × 19 mm, I.D., 5 μm particle size, Waters Corp., MA, USA) at the flow rate of 12.0 mL/min by the elution of formic acid/H_2_O/acetonitrile (0.1/55/45 in *v/v/v*) using an HPLC apparatus (PU-980 HPLC Pump, JASCO Corp., Tokyo, Japan), then the yield (0.029%) of 5.8 mg pteryxin was purified.

### 2.3. Analysis of Pteryxin

The structure of pteryxin was determined by its ^1^H and ^13^C-NMR spectra (Avance III HD Ascend 400 MHz spectrometer, Bruker Billerica, MA, USA), and its molecular formula was determined by Q-TOF LC/MS (Agilent 6530 Accurate-Mass Q-TOF LC/MS system Agilent Technologies, CA, USA) on a reversed-phase chromatographic column (ACQUITY UPLC BEH C18, 50 × 2.1 mm I.D., 1.7 μM particle size, Waters Corp., MA, USA) at 40 °C. The mobile phase consisting of a 0.1% formic acid aqueous solution / 0.1% formic acid containing acetonitrile (1:1) was carried out at the flow rate of 0.4 mL/min by a linear gradient to 0.1% formic acid aqueous solution/0.1% formic acid containing acetonitrile (1:19) at 3 min. The high-resolution mass spectra (HRMS) was measured under the following conditions: a positive ion mode; a desolvation temperature, 350 °C; a desolvation pressure, 40 psig; and a desolvation gas flow, 8 L/min.

### 2.4. Cell Culture

RAW264.7 cells (mouse macrophages) were obtained from American Type Culture Collection (VA, USA). Cells were cultured in DMEM medium (including 10 % FBS, 100 U/mL penicillin and 100 µg/mL streptomycin) at 37 °C in a 5% CO_2_ atmosphere.

### 2.5. Cell Survaival

The cell viability treatment, with or without a test sample in a well, was examined by an MTT assay, as previously reported [16]. After the culture, MTT (0.05%) was added to each well and incubated for 3 h. The formazan reduced from an MTT was extracted with DMSO (100 µL) and determined as an index of the surviving cells at 570 nm using a microplate reader (BIO-RAD Model 550, BIO-RAD, CA, USA).

### 2.6. Activity of Nrf2-ARE Signaling

The activity of Nrf2-ARE signal treatment, with or without a test sample, was examined by a reporter assay, as previously reported (14,15). The RAW264.7 cells, with or without the test sample, were pre-cultured on a 12-well microplate (5 × 10^5^ cells/well) for 24 h, and then the pGL4.37 [luc/ARE/Hygro] plasmid and VIOFECTINE (as the transfection reagent) were co-transfected in cells. After 24 h, the cells were washed twice in PBS and lysed in 100 µL lysis buffer. The luciferase activity of the lysate cells (50 µL) was assayed using a luciferase substrate, then the chemiluminescence (CL) in cells was measured by a microplate reader (GLOMAX MULTI Detection system, Promega, WI, USA). The protein concentration of the cells was determined using a BCA protein assay kit (Thermo Fisher Scientific, Inc., MA, USA).

### 2.7. Nuclear Extraction and Determination of Nrf2

The Nrf2 translocation in nucleus was examined as previously reported (14). Cells (1.0 × 10^6^ cells/mL) with or without the compound (50 and 100 μM) were incubated for 24 h. After 24 h, the cells were treated with trypsin. The nucleus of the cells was extracted using an extraction kit (NE-PER Nuclear and Cytoplasmic Extraction Reagent, Thermo Fisher Scientific K.K, Yokohama, Japan). Nrf2 protein was detected using anti-Nrf2 by a Western blot analysis, and the Nrf2 protein expression was expressed as % of the untreated sample cells (control).

### 2.8. Protein Expression by Western Blot Analysis

The assessed cells were treated with the lysis buffer. The cellular lysates were centrifuged at 13,800 *g* for 5 min. The cellular extracts were separated on SDS-polyacrylamide gels (4–12% SDS-polyacrylamide, Invitrogen, CA, USA) and transferred to a nitrocellulose membrane (iBlot Gel Transfer Mini, Invitrogen) using an iBlot Gel Transfer Device (Invitrogen). The protein was detected with the antibodies, such as Nrf2 and HO-1, and the protein expression was determined by densitometry analysis.

## 3. Results

### 3.1. Determination of Pteryxin

The chemical structure of (+)-pteryxin ([α]${}_{D}^{24}$ = 10.9° (*c* 0.13, EtOH)) was determined by the following ^1^H and ^13^C NMR spectra. The ^1^H NMR (400 MHz, CDCl_3_, δ_H_ 7.26): 7.59 (1H, d, *J* = 9.5 Hz, H-4), 7.35 (1H, d, *J* = 8.6 Hz, H-5), 6.80 (1H, d, *J* = 8.6 Hz, H-6), 6.63 (1H, d, *J* = 5.0 Hz, H-4′), 6.22 (1H, d, *J* = 9.5 Hz, H-3), 6.03 (1H, qq, *J* = 7.2, 1.5 Hz, H-3″), 5.35 (1H, d, *J* = 5.0 Hz, H-3′), 2.09 (3H, s, OCOCH_3_), 2.00 (3H, dq, *J* = 7.2, 1.5 Hz, H-4″), 1.86 (3H, dq, *J* = 4.5, 1.5 Hz, H-5″), 1.46 (3H, s, 2′-CH_3_), 1.43 (3H, s, 2′-CH3). ^13^C NMR (100 MHz, CDCl_3_, δ_C_ 77.0): δ_C_ 169.8 (OCOCH_3_), 166.9 (C-1″), 159.7 (C-2), 156.6 (C-7), 154.0 (C-9), 143.1 (C-4), 137.9 (C-3″), 129.2 (C-5), 127.4 (C-2″), 114.4 (C-6), 113.3 (C-3), 112.5 (C-10), 107.3 (C-8), 77.3 (C-2′), 70.5 (C-3′), 60.1 (C-4′), 25.3 (2′-CH_3_), 22.2 (2′-CH_3_), 20.7 (OCOCH_3_), 20.4 (C-5″), 15.6 (C-4′). 

**Figure 1:**
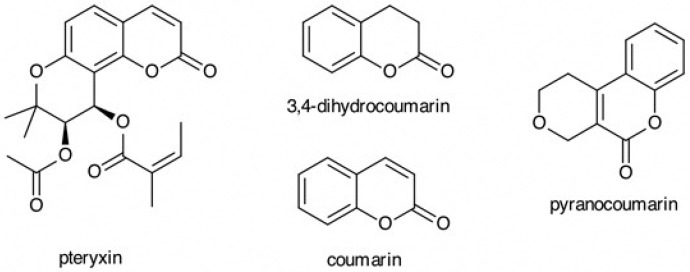
Chemical structures of pteryxin and the derivatives used in this study.

In addition, the molecular formula was established as C_21_H_22_O_7_ on the basis of HRMS (*m/z* 387.1448 [M+H]^+^, calcd. 387.1438, and *m/z* 409.1262 [M+Na]^+^, calcd. 409.1258). The chemical structure of pteryxin is shown in Figure 1. Pteryxin is an angular-type khellacton coumarin substituted acyl groups. In this study, the Nrf2 activity of the major types of coumarins, such as angular type pyranocoumarin, simple coumarin, and its dihydotype coumarin (3,4-dihydrocoumarin), were assessed together with the pteryxin (Figure 1).

### 3.2. Nrf2-ARE Signaling

**Figure 2:**
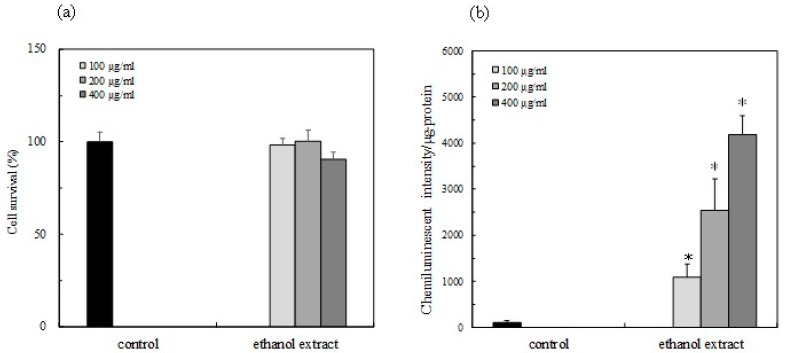
Activation of the Nrf2 (Nuclear factor-erythroid-2-related factor)-ARE (antioxidant response element) signaling in the presence of the ethanol extract of *Peucedanum japonicum* Thunb leaves in RAW264.7 macrophage cells. (**a**) Cell viability with treated samples at the test concentrations was examined by an MTT assay. The cell viability was expressed as % of the control cells without sample. The Nrf2-ARE signaling activity of the EtOH extract of the *P. japonicum* leaves was evaluated by the reporter assay as described in the text. (**b**) The effect of the various concentrations (100–400 μg/mL) of the EtOH extract of *P. japonicum* leaves in RAW264.7 cells. The activity (%) was indicated as % of induction for the control cells without a sample. Data were expressed as mean ± SD, and the significant difference was analyzed by the student’s *t*-test. * *p* < 0.01 indicated as a significant difference from the control.

*P. japonicum* leaves were extracted with ethanol, and the cytotoxicity of the extract was evaluated by an MTT assay. As shown in Figure 2a, the cytotoxicity of the extract was not detected in the range of the test concentrations. The various concentrations (100, 200 and 400 μg/mL) of the extract were evaluated for the activity of the Nrf2-ARE signaling in RAW264.7 cells (Figure 2b). The extract was significantly activated in a dose-dependent manner, suggesting that the Nrf2 activator is contained in the extract (Figure 2b).

**Figure 3:**
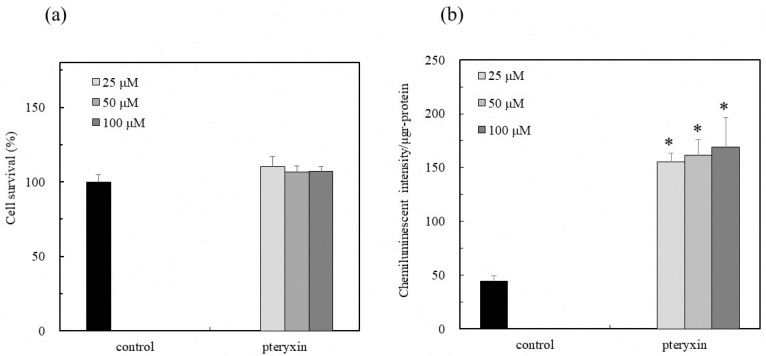
Nrf2-ARE signaling activity due to pteryxin in RAW264.7 macrophage cells. The Nrf2-ARE signaling activity in the presence of pteryxin (25–100 µM) was evaluated by the reporter assay, as described in the text. (**a**) Cell viability with treated samples at the test concentrations was examined by an MTT assay. The cell viability was expressed as % of control cells without a sample. (**b**) The effect of the various concentrations (25–100 µM) of pteryxin in RAW264.7 cells. The activity was indicated as induction (%) for control cells without sample. Data were expressed as mean ± SD, and the significant difference was analyzed by the student’s *t*-test. * *p* < 0.01 indicated a significant difference from the control.

Pteryxin was isolated from the ethanol extract of *P. japonicum* leaves. As shown in Figure 3a, the cytotoxicity of pteryxin was not detected in the range of the test concentrations. When pteryxin was placed in the Nrf2-ARE signaling cell system, the Nrf2 activity was significantly induced in the concentration range of 25–100 µM, indicating that pteryxin is one of the active compounds in the *P. japonicum* leaves (Figure 3b).

### 3.3. Nrf2 Expression in Cytoplasm and Nuclei

**Figure 4:**
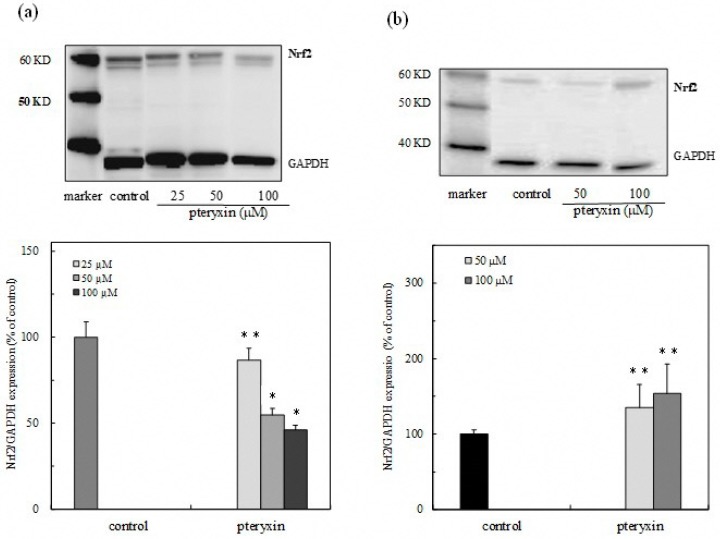
Expression of the transcription factor Nrf2 protein due to pteryxin in RAW 264.7 macrophage cells. The Nrf2 protein expression in the presence of a compound was detected by western blot analysis and determined by densitometry. (**a**) Cytoplasmic Nrf2 protein expression and (**b**) nuclear Nrf2 protein expression in the presence of a compound. Data were expressed as mean ± SD, and the significant difference was analyzed by the student’s *t*-test. * *p* < 0.01 and ** *p* < 0.05 indicated a significant difference from the control.

The expression of cytoplasmic Nrf2 in the presence of a compound was determined by Western blot analysis. The Nrf2 was accumulated in the cytoplasm in a dose-dependent manner, and the accumulation of the transcription factor Nrf2 was also detected in the nucleus, suggesting that the cytoplasmic Nrf2 translocated into the nucleus through the Nrf2-ARE signaling (Figure 4a,b). Consequently, the transcription factor Nrf2 would be activated on the ARE regions, resulting in the expression of the HO-1 protein.

### 3.4. HO-1 Expression

**Figure 5:**
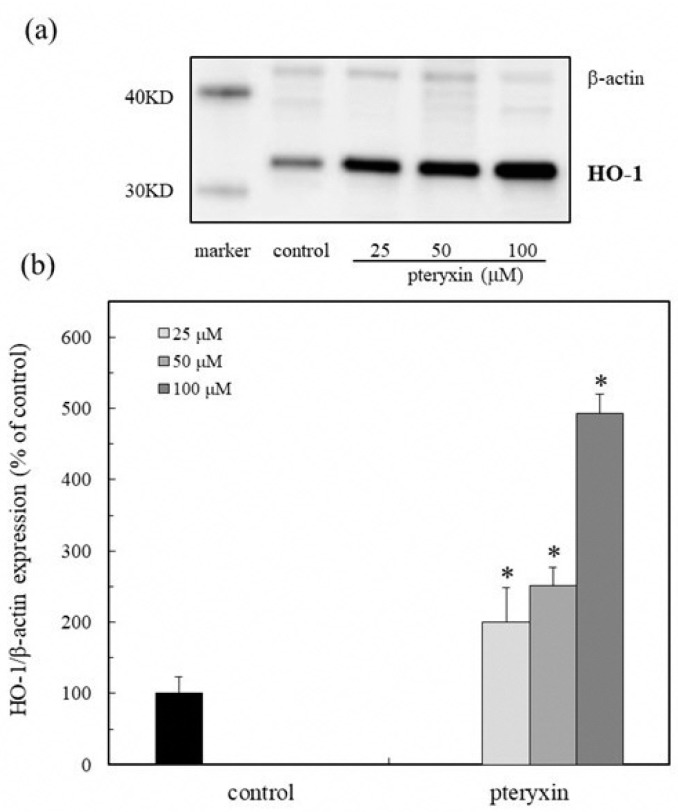
HO-1 (heme oxygenase-1) expression due to pteryxin in RAW 264.7 macrophage cells. The HO-1 protein expression due to pteryxin (25–100 µM) on the Nrf2-ARE signaling in the cells was examined. (**a**) Western blot analysis of the HO-1 protein in the presence of a compound. (**b**) Densitometry analysis of the expression of the HO-1 protein. Data were expressed as mean ± SD, and the significant difference was analyzed by the student’s *t*-test. * *p* < 0.01 indicated a significant difference from the control.

The expression of the antioxidant protein HO-1 in the presence of the target compound was determined by Western blot analysis. The protein expression increased in the concentration range of 25–100 μM (Figure 5a,b). Consequently, the Nrf2 in the nucleus was enhanced by the HO-1 protein expression (Figure 4). This result indicates that the pteryxin plays a significant role in delaying the oxidative stress in a biological system. 

### 3.5. Nrf2-ARE Signaling Activity and HO-1 Protein Expression by Coumarin Derivatives

**Figure 6:**
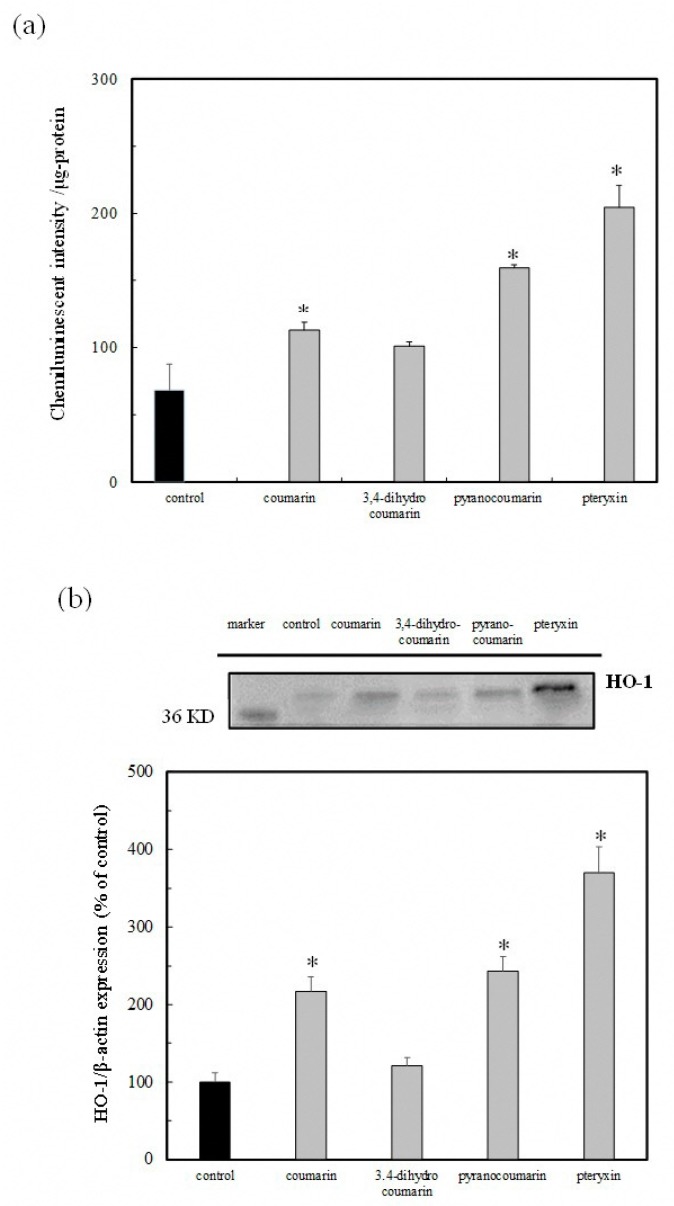
Activation of Nrf2-ARE signaling and the HO-1 protein expression due to coumarin derivatives in the RAW 264.7 macrophage cells. (**a**) The activation of Nrf2-ARE signaling due to the coumarin derivatives (50 µM) was assessed by the reporter assay. (**b**) The HO-1 protein expression due to the various coumarins (50 µM) was detected by Western blot analysis. The expression of the HO-1 protein was determined by a densitometry analysis. Data were expressed as mean ± SD, and the significant difference was analyzed by the student’s *t*-test. * *p* < 0.01 indicated a significant difference from the control.

The Nrf2-ARE signaling activity of pteryxin was compared to the other types of coumarins, such as simple coumarin, 3,4-dihydrocoumarin, and pyranocoumarin. The Nrf2 activity of these compounds was determined by the reporter assay (Figure 6a). As a result, the Nrf2 activity was detected by the α,β−carbonyl coumarins, except for 3,4-dihydrocoumarin, which suggested that the electrophyllicity in the molecule contributes to the cysteine thiol oxidation of Keap1 and leads to the activation of the Nrf2-ARE signaling. Particularly, the activity of pteryxin, which was the highest of the evaluated coumarin derivatives, indicated that the structure of khellacton is suitable for the electrophyllicity.

The HO-1 protein expression, due to the structurally different coumarins, was assessed together with pteryxin. The pteryxin and pyranocoumarin, in common with an angular-type skeleton, presented a high expression, which had a similar result to that of the Nrf2 activity (Figure 6b). Particularly, the pteryxin, which consisted of khellacton-substituted acyl groups, had the highest Nrf2 activity involving the HO-1 protein expression. These results suggest that the difference in the Nrf2 activity due to the compounds may be dependent on the individual potential electrophyllicity.

## 4. Discussion

*Peucedanum* species are used as a traditional medicine for sore throats, coughs, colds, and headaches [1]. Recent studies have demonstrated that the extract of *P. japonicum* Thunb plays a role in suppressing obesity. Particularly, some coumarins in the *Peucedanum* species have been examined based on their anti-diabetes and anti-obesity activities [4,7,8]. In addition, several anti-obesity components, including pteryxin and the other coumarin derivatives, were found in *P. japonicum* Thunb, and they were mainly exerted by inhibition of lipogenesis in the adypocytes.

A more recent study indicated that the oxidative stress in the hypothalamus induces insulin resistance and obesity. The Nrf2 activity then suppressed the hypothalamic oxidative stress, subsequently improving the resistance of insulin and leptin related to obesity [17]. This study demonstrated that the leaf extract of *P. japonicum* Thunb has the physiological function of activating the Nrf2-ARE signaling to avoid cell damage by the excess production of the reactive oxygen species (ROS) under oxidative stress (Figure 2). In addition, the pteryxin, as one of the main Nrf2 activators in the extract, dissociated the Nrf2 from Keap1, and then the Nrf2 translocated into the nucleus, activated the ARE region containing the promoter and enhancer regions-mediated antioxidant enzyme, HO-1 (Figure 3, Figure 4 and Figure 5). Some α,β carbonyls in the molecule will be potential electrophiles that react with the nucleophile protein, Keap1 [18]. When the cysteine residue in the Keap1 is oxidized by an electrophile, the Nrf2 part from Keap1 binds to the ARE region in the DNA sequences. Our results, together with previous knowledge, suggest that the coumarins that are effective against diabetes mellitus and obesity may also act in conjunction with the Nrf2 activity [8]. 

Coumarins are a large class of plant secondary compounds with a benzopyrone skeleton; they are distributed across four major sub-types: simple coumarins, furanocoumarins, pyran-substituted coumarins, and pyranocoumarins. The pteryxin is an angular-type pyranocoumarin (khellacton coumarin) substituted acyl group (Figure 1). In this study, the Nrf2 activity of the structurally different coumarins—angular-type pyranocoumarin, simple coumarin, and 3,4-dihydrocoumari— were examined together with the pteryxin, and the activation of the Nrf2 activity was detected by the α,β carbonyl coumarins, except for 3,4-dihydrocoumarin (Figure 6a). The HO-1 expression was also similar to the result of the Nrf2 signaling activity (Figure 6b). A previous study demonstrated that the α,β carbonyl in 1,2-naphtoquintione is an electrophile that resulted in the nucleophile protein, Keap1, which is added to the carbon β by Michael addition [18]. A similar result was obtained, except for 3,4-dihydrocoumarin, which suggested that the α,β carbonyl in the molecule plays a significant role as an electrophile for the Nrf2 activator. Also, pteryxin and pyranocoumarin indicated a high Nrf2 activity, which suggested that the angular skeleton will be an effective structure for an Nrf2 activator. In addition, the pteryxin, consisting of khellacton-substituted acyl groups, indicated the highest Nrf2 activity involving HO-1 expression, suggesting that the acyl groups may also contribute to the electrophyllicity in the molecule. 

Choi et al. reported the anti-adipogenic and anti-diabetic effects of cis-3,4-diisovalerylkhellactone isolated from *P. japonicum* Thunb [8]. This khellacton coumarin substituted acyl group has a structure similar to that of pteryxin, which may have potential Nrf2 activity. In a previous study, the effect anti-obesity effect due to pteryxin was elucidated through animal testing [14]. The Nrf2 plays a significant role in the regulation of obesity and insulin resistance [11]. The Nrf2 activity suppressed the hypothalamic oxidative stress, resulting in the improvement of the resistance of insulin and leptin related to obesity [17]. Thus, the Nrf2 active function will play a key role in prohibiting obesity and diabetes mellitus. In addition, pteryxin will be a useful agent for functional foods, preventing the metabolic syndrome based on insulin resistance.

## 5. Conclusions

In this study, the highest Nrf2 activity was found in EtOH extract of *P. japonicum* leaves, and its Nrf2 active compound was identical to that of pteryxin. The accumulation of the Nrf2 in the nucleus due to pteryxin induced the expression of the antioxidant protein, HO-1. In addition, the Nrf2 active function, due to pteryxin, was suggested to hold electrophillicity due to the α,β-carbonyl and/or substituted acyl groups in the molecule modulating the dissociation of Nrf2 from the Keap 1.
